# Museum of Comparative Zoology

**Published:** 1861-01

**Authors:** 


MUSEUM OF COMPARATIVE ZOOLOGY.—Owing to the indomitable energy of Professor Agassiz, the curator and director of the new institution, within two years a fund of $225,000 and a large number of specimens have been collected, besides five acres of land given by Harvard University, with which the museum went into operation, on the thirteenth of November. It is connected with the University, and at the inauguration Professor Agassiz stated, "we have already outrun all the museums of the European Universities, excepting those placed in large capitals, and among these we would probably occupy the ninth or tenth place."

